# A Novel Adaptive Platform for Rapid, Simple Flow-Based Antibody Detection Devices Predicts NAb Levels to SARS-CoV-2

**DOI:** 10.20411/pai.v11i1.910

**Published:** 2026-02-16

**Authors:** Alena J. Markmann, D. Ryan Bhowmik, Baowei Jiang, Usaphea P. Vanna, Michael Van Hoy, Frank Wang, Yixuan J. Hou, David M. Margolis, Ralph S. Baric, Aravinda M. de Silva, Luther A. Bartelt

**Affiliations:** 1 Department of Medicine, Division of Infectious Diseases, University of North Carolina School of Medicine, Chapel Hill, North Carolina; 2 Department of Microbiology and Immunology, University of North Carolina School of Medicine, Chapel Hill, North Carolina; 3 Current affiliation: Institute of Bioinformatics, University of Georgia, Athens, Georgia; 4 BioMedomics Inc., Morrisville, North Carolina; 5 Department of Pharmacology, University of North Carolina at Chapel Hill, North Carolina; 6 Current affiliation: Transformative Legal, LLC., Scottsdale, Arizona; 7 Department of Epidemiology, School of Public Health, University of North Carolina, Chapel Hill, North Carolina; 8 Current affiliation: Moderna Therapeutics Inc., Cambridge, Massachusetts; 9 UNC HIV Cure Center, University of North Carolina School of Medicine, Chapel Hill, North Carolina

**Keywords:** Rapid Diagnostic, COVID-19 Immunity, SARS-CoV-2, Antibody Response, Neutralizing Antibodies, Correlate of Immune Protection

## Abstract

**Background:**

COVID-19 has caused millions of deaths and continues to burden individuals and the healthcare system. Antibodies that neutralize SARS-CoV-2 have proven to be the most reliable markers of immune protection, targets for vaccine development, and approaches for anti-viral antibody-based therapies. Measuring neutralizing antibody (NAb) titers at the bedside could inform individualized shared decision-making with patients regarding the potential benefits of repeating vaccines, use of preventative or therapeutic antibody-based therapies, and, where relevant, collection of COVID-19 convalescent plasma (CCP) with greater efficacy, especially as NAb-escape mutations have guided SARS-CoV-2 variant emergence. However, specific and accessible assays to quantify NAb levels in individuals, including the identification of potential antibody donors at the time of donation, remain unavailable. Therefore, there is a need for platforms that can be rapidly adapted to quantify serum antibody responses with known or expected correlates of protection.

**Methods:**

In this report, we apply a novel semi-quantitative method to an established antibody lateral flow assay (sqLFA) and analyze its ability to detect the presence of functional NAbs in the serum of COVID-19-recovered individuals early in the pandemic.

**Results:**

We found that the sqLFA has a strong positive correlation with the gold-standard microneutralization assay (specificity 80% and sensitivity 90% at a microneutralization cutoff of 1:40).

**Conclusions:**

Taken together, the sqLFA provides a novel point-of-care-based platform for rapid readout of NAb-based immune protection to SARS-CoV-2.

## INTRODUCTION

Quantitative antibody detection platforms can assess an individual’s antibody immune response. Rapid and accessible platforms for these assays can be highly valuable at the beginning of a viral epidemic or pandemic for a range of vaccine-preventable infections. Viral infections generate antibody responses, and neutralizing antibody (NAb) responses have been found to be correlates of immune protection (CoP) for many viruses, including SARS-CoV-2 [1]. Despite rapid progress in the SARS-CoV-2 field, the now-normal periodic circulation of novel variants each year and diminishing efficacy of antibody-based therapies have led to the arrested development of rapid point-of-care antibody testing that could help identify those at particular risk of symptomatic infection. These assays can measure various antibody subclasses, including IgG, IgM, and IgA, as well as combinations of these. Clinical lab-based antibody-binding assays, such as enzyme-linked immunosorbent assays (ELISAs) [2], have been developed after being found to provide a satisfactory correlate of protection for some infections such as Hepatitis A and B, *Haemophilus influenzae*, pertussis, pneumococcus, and tick-borne encephalitis [3]. The most extensively studied antibody immune correlate of protection is for Hepatitis B, where vaccine efficacy studies were performed alongside antibody titer measurements in longitudinal vaccinated cohorts [4]. These studies provide guidance on how antibody CoP could be implemented, but none compare antibody levels to rapid point-of-care testing or provide guidance on the accuracy of rapid antibody testing for CoP.

Lateral flow assays (LFA) are rapid point-of-care antibody-based binding assays that can be performed at home or easily in a clinic, unlike labor-intensive ELISAs, which are usually performed in Clinical Laboratory Improvement Amendent (CLIA)-certified clinical laboratories. Direct neutralization assays are not routinely performed in CLIA-certified clinical laboratories because these labor-intensive assays require BSL3 conditions and are therefore generally restricted to health departments, the Centers for Disease Control and Prevention, or in non-CLIA-certified research laboratories. NAb levels have been identified as a strong correlate of protection for COVID-19, the disease caused by SARS-CoV-2 [2, 5–7]. The immunodominant epitope on the spike envelope protein in SARS-CoV-2 is the receptor-binding domain (RBD), which is the target of 90% of the SARS-CoV-2 NAb response [8]. Antibodies that can bind the RBD can effectively block the virus’s ability to attach to and to enter human epithelial and endothelial cells. In a recent study, researchers found that D614G variant NAbs provided 37% of total protection against delta wave infection at a titer >1:67 [6]. However, NAb responses to natural SARS-CoV-2 infection and vaccination depend on prior variant exposure and are highly variable between individuals in both titer and durability [9–13]. In a recent vaccine efficacy household study with 1461 participants, a NAb titer to Wuhan SARS-CoV-2 strains ≥1:1024 was necessary to confer 92% protection against infection from a delta variant [14]. In a modeling study that used data from 7 vaccine trials and 246 individuals, a NAb titer of 1:54 IU/mL was 50% protective against infection [15]. Clinical trials evaluating antibody-based therapies for COVID-19 have identified a clear benefit to participants who are enrolled prior to antibody seroconversion [16], highlighting the importance that antibody therapies play in early disease.

Additional roles for NAbs as a correlate of clinical outcomes can be drawn from studies of convalescent plasma (CCP). Early in a pandemic, there are often few therapies, and CCP from recovered individuals is often used because it contains antibodies to the novel organism. CCP studies have concluded that it was and continues to be beneficial, especially in immunocompromised populations, if the NAb titer is adequately high (>1:40) and if it is infused early after illness onset [17–21]. However, commercially available rapid antibody detection tests developed during the first year of the pandemic were not rigorously tested against NAb assays to understand whether they correlated with immune protection, and in outcomes-based trials, they were less predictive of CCP efficacy than direct NAb testing [15, 22].

Rapid quantitative antibody assays have dual use: they can be used to rationally design vaccine schedules, particularly for individuals who need repeated vaccination to sustain adequate NAb titers to avoid breakthrough infections, and to identify those who qualify to donate antibody-based therapies in settings where other therapies are unavailable, and in the context of newly emerging variants [13]. For antibody-based assays to inform clinical decisions and public health interventions, quantitative antibody assays need to be developed and validated against more rigorous functional antibody assays, and the technology to detect these antibodies must be widely accessible [23, 24]. Similarly, to progress studies of precise estimates of markers of CoP, the assays or correlations need to be affordable and widely accessible to the community of investigators.

In this study, we use a large cohort of individuals naturally infected with the original SARS-CoV-2 strains and free of interference from prior vaccinations or repeated infections to evaluate a previously clinically validated simple lateral flow-based device [25] in a new prototype reader system that provides a semi-quantitative lateral flow assay (sqLFA), and we correlate the results with the gold-standard NAb titers [26]. We show that our novel platform can be used with a simple lateral flow device to distinguish individuals with high NAb levels from those with lower NAb levels with good sensitivity and specificity. We also find that the sqLFA readout correlates better with NAb levels than other CLIA-certified SARS-CoV-2 antibody assays. This semi-quantitative platform, completed within 20 minutes, can be adapted to various screening antigens rapidly in the setting of a new epidemic or pandemic, as new viral variants emerge, and as CoP data refines rationales for vaccine schedules and antibody-based therapies at the individual patient level.

## METHODS

### Study Samples

This study was conducted under Good Clinical Research Practices and was compliant with Institutional Review Board oversight approved by the UNC IRB (20-1141); consent was obtained from all participants. A total of 268 convalescent SARS-CoV-2 plasma samples from patients with natural pre-delta variant infection prior to any vaccination availability were used for this investigation. These samples were collected at a median of 62 days following polymerase chain reaction diagnosis (n = 215) or symptom onset (n = 53), whichever came first, with a range of 12–337 days. Prior characterization of this cohort has been published [11]. Different numbers of the same samples were run in our various antibody assays, and that information is shown in Supplementary Table 1.

### BioMedomics sqLFA System

**Figure 1.**
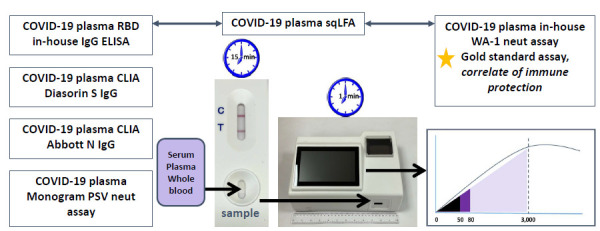
**Outline of assays performed.** All assays were compared to and correlated with each other as well as with gold-standard assay: WA-1 live virus neutralization assay. Workflow of sqLFA sample application, insertion into prototype reader, and RI readout. 0-50 RI = negative (black), 50-80 = indeterminate (dark purple), >80 = positive (light purple). Abbreviations: RI, reflective intensity; sqLFA, semi-quantitative lateral flow assay.

To detect RBD-binding IgG antibodies, we applied a BioMedomics LFA test, which has been separately validated as a rapid diagnostic test to detect SARS-CoV-2 RBD-binding antibodies [25]. The BioMedomics LFA has also been previously validated for whole blood as well as different types of venous samples, including plasma [25]. In the current assay, 10 µL of serum or plasma from each sample was pipetted onto the BioMedomics LFA IgG test strip, followed by 3 drops of buffer solution provided with the kit per manufacturer’s instructions. Each LFA strip was developed for 13 to 15 minutes at room temperature with standard lighting conditions (Figure 1). The LFA strip was then inserted into a prototype reader (Figure 1), which displayed both a qualitative result (Positive/Negative/Intermediate) and a quantitative result in the form of reflective intensity (RI) of gold particles on the LFA strip, which ranged from 0 to 3000. Values were positive according to the manufacturer’s protocol: positive: RI >80, intermediate: RI 50-80, and negative: RI <50. To determine linearity of the RI detector, human IgG antibody was diluted in human serum at different concentrations. Each sample was then tested on the previously validated BioMedomics IgG RBD LFA and read by the prototype reader. The data was then used to create a calibration curve for the detector.

### Other SARS-CoV-2 Antibody Assays

Results from the sqLFA were compared to an in-house SARS-CoV-2 RBD IgG ELISA (end-point-titer), a CLIA-certified lab-run nucleocapsid IgG ELISA (Abbott), a CLIA-certified lab-run full-length Spike IgG ELISA (DiaSorin) [11, 26], and a live virus luciferase reporter-based functional neutralization assay with a readout of 50% neutralization of viral infection (NT50) [26]. The DiaSorin was approved by the Food and Drug Administration (FDA) in 2021 as “Acceptable for Use in the Manufacture of COVID-19 Convalescent Plasma with High Titers of Anti-SARS-CoV-2 Antibodies” at a cutoff of ≥ 87 AU/mL [27]. These assays are listed in Figure 1 and were all performed as previously described [11].

### Statistics

All statistical analyses and graphs were generated using GraphPad Prism 10.3.1 for Windows [28]. A non-parametric Spearman correlation coefficient was calculated using GraphPad Prism to compare the BioMedomics sqLFA RI to the NT50 titer and other antibody-binding ELISA assays such as our in-house RBD IgG ELISA quantitative end-point titer. All tests were 2-tailed, and a *P*-value less than 0.05% was considered statistically significant. Receiver operating characteristics (ROC) curves [29] were conducted to determine the sensitivity and specificity of the BioMedomics sqLFA in detection of NT50 at various NAb titer cutoffs. Youden’s J statistic was calculated for each ROC curve and used to maximize both sensitivity and specificity, as presented here. Correlation plots were further analyzed using simple linear regression, with the 95% confidence intervals of the best-fit line shown and shaded in grey.

## RESULTS

### Evaluation of BioMedomics sqLFA Platform in Measuring NAb Titer

**Figure 2.**
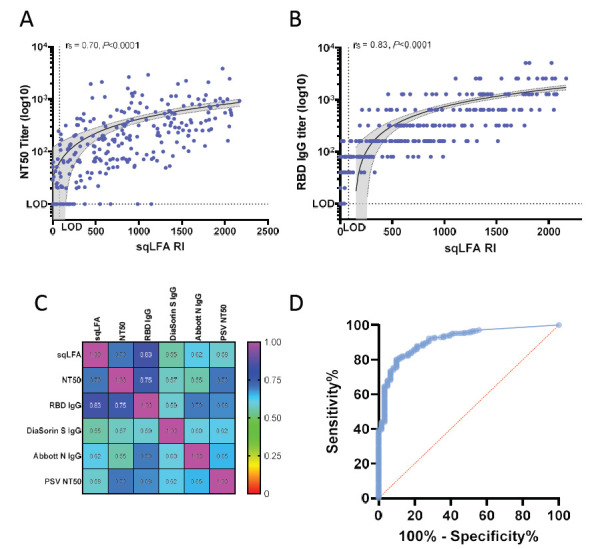
**Correlation plots between sqLFA and other serologic assays.** A) WA-1 neutralization assays correlation plot with sqLFA. B) RBD IgG in-house ELISA correlation plot with sqLFA, grey shaded area is 95% CI for simple linear regression analysis. C) Heat map of correlation plots for all assays performed with the same samples. D) Example ROC curve of sqLFA performance for all samples with NT50 titer >1:40. Abbreviations: CI, confidence interval; ELISA, enzyme-linked immunosorbent assay; RBD, receptor binding domain; ROC, receiver operating characteristics curve; sqLFA, semi-quantitative lateral flow assay

We found that the sqLFA RI readout for the samples tested ranged from 0 to 2169 and was positively correlated with NT50 titers (Figure 2A), with a Spearman correlation coefficient (r_s_) of 0.70 (*P* < 0.0001, n = 268). We also obtained r_s_ for samples that were 14-59 days, ≥ 60 days, or ≥ 90 days post diagnosis or symptom onset; in all cases, r_s_ = 0.70 (*P* < 0.0001) (data not shown), so within our sample range, the timing post-infection did not change this strong correlation. Among 38 randomly selected samples repeatedly tested on different days (median NT50 titer = 1054, range 0-2076), the intra-assay variability Spearman correlation was r_s_ = 0.94 (*P* < 0.0001).

Our prior work identified that SARS-CoV-2 RBD antibody binding in an in-house assay was a better correlate of NAb NT50 than N-terminal domain, full-length spike protein, and nucleocapsid antigens [11]. Compared to the in-house quantitative RBD IgG ELISA end-point titer data, the sqLFA RI values correlated positively (r_s_ = 0.83; *P* < 0.0001) (Figure 2B). Among 94 samples tested, the sqLFA correlated modestly with the DiaSorin trimeric spike IgG assay (r_s_ = 0.55; *P* < 0.0001) (Figure 2C), which is significantly lower than sqLFA vs NT50. Correlation with a commercial SARS-CoV-2 nucleocapsid IgG (Abbott) was even lower at r_s_ = 0.38 (Figure 2C). These findings are consistent with known limitations in both spike and nucleocapsid based IgG binding assays that are not tightly correlated with NT50. Turnaround times for results from these assays were 15 minutes for the sqLFA, 3-6 hours for binding assays (RBD, DiaSorin, Abbott), and a minimum of 72 hours for the live virus NT50.

### Sensitivity and Specificity Analyses

There is no accepted guideline-directed NAb titer target as a correlate of protection, and consequently, desirable sensitivity and specificity performance beyond diagnosis of exposure is unavailable. We therefore used the literature, including published clinical protocols, to perform analyses using theoretical cutoffs for different NAb titer goals across scenarios. Vaccine breakthrough infection studies, for example, have identified vaccine efficacy and NAb NT50 titer ranges of 1:40, 1:54, and up to 1:1024, depending upon the variant of interest and specific NT50 standard assay used. The original FDA-recommended threshold for therapeutic applications of CCP infusion was NT50 >1:160, and an NT50 >1:640 is an often-cited cutoff for “high titer” serum or plasma samples [30] with predicted greater efficacy.

Multiple ROC analyses were performed to calculate the sensitivity and specificity of the sqLFA RI for detecting samples at different levels of functional Nabs (Table 1). Samples that had detectable NAb titers (NT50 ≥1:40 or other higher cutoffs) were set as positive controls, and those with undetectable NAb titers (or below the cutoff) were set as negative controls using data from our in-house NT50 assay. The area under the curve (AUC) for the sqLFA to detect any NAb NT50 ≥1:40 was 93%, *P* < 0.0001 (Table 1, Figure 1D). The sensitivity increased to 97% for detecting samples with NT50 ≥1:40 when using the manufacturer’s cutoff of RI > 80. The specificity at this cutoff was low at 46%, which is an expected drop-off at lower titers where functional neutralization is known to be more variable between individuals. The threshold sqLFA cutoff to achieve a specificity >90% for an NT50 ≥1:40 was RI > 457. At an NT50 neutralizing antibody titer of ≥1:54, identified by Khoury et al as a key correlate of 50% protection following vaccination and arguably the most relevant efficacy analysis to date [15], our sqLFA achieved an AUC of 0.85 (*P* < 0.0001), with both sensitivity and specificity of 82% (Table 1).

**Table 1.** Sensitivity and Specificity of qLFA for Detecting Neutralizing Antibodies

qLFA cutoff	AUC	Sensitivity (%)	95% Cl	Specificity (%)	95% Cl
**NT50 ≥ 1:40** >457	0.93, p<0.0001	80	74.2–85.0	90	80.2–95.4
**NT50 ≥ 1:54** >457	0.89, p<0.0001	82	75.5–86.4	82	72.0–89.3
**NT50 ≥ 1:1024** >872	0.71, p=0.0016	80	58.4–91.9	62	55.5–67.5

ROC analysis results for each NT50 cutoff, showing the sqLFA cutoff with the best Youden’s J statistic to maximize both Sensitivity and Specificity. 95% CI = 95% confidence intervals.

We found that at higher NT50 NAb titer cutoffs, the sqLFA had both a high specificity and sensitivity.

## DISCUSSION

To our knowledge, this is the first study that compares a validated LFA point-of-care test with a semi-quantitative reader platform to a newly published quantitative correlate of protection against SARS-CoV-2 infection, NAb NT50 titers. Here, we demonstrate that the BioMedomics sqLFA rapid platform can serve as a sensitive tool for detecting protective levels of neutralizing antibodies in human samples, providing a proof of concept for future studies and future development of clinically relevant serology-based diagnostics, an area of critical need for respiratory viral infections. From prior work, we know that the immunodominant target on the SARS-CoV-2 spike protein for NAbs is the RBD [2]. The sqLFA platform system with the SARS-CoV-2 RBD antigen has a strong positive correlation with NT50 NAb titers and can be used at various cutoffs to detect different NAb NT50 levels depending on the application. Furthermore, it is easy to use and can be adapted within a few weeks to novel antigens in the event of virus escape mutations or future pandemics, such as the current threat of avian influenza [31]. There is an ongoing need for rapid, deployable tools to assess SARS-CoV-2 immunity post-natural infection or vaccination, especially in immune-susceptible populations. For example, as a future direction, we envision the possibility of generating an LFA with multiple antigens on a single strip to represent current circulating strains as well as vaccine antigens and validating it for use with whole venous blood and potentially fingerstick blood samples.

Our study showed that at an RI > 872, the BioMedomics sqLFA specifically detects NAb levels ≥ 1:1024, which have very recently been shown in 2 separate studies to be a strong CoP against symptomatic alpha or delta strain infection [13, 14, 32]. This correlation far exceeds commercially available spike and nucleocapsid IgG assays available in our clinical laboratory. Using this platform, it is possible to consider a range of patient/individual-directed applications such as timing for repeat COVID-19 vaccine booster (especially in the context of periodic endemic natural infection), need for passive antibody-based therapies in vaccine non-responders, and identification and recruitment of individuals to donate high-titer CCP if such situations arise. The sqLFA point-of-care platform also has a small footprint (Figure 1), low intra-assay variability, and takes less than 20 minutes to set up and obtain a result, making it a great candidate for clinical use and for resource-restricted areas such as much of the rural United States.

Other rapid, semi-quantitative assays have been published and also found to positively correlate with NAb levels, supporting our results here [24, 29, 33, 34]. Two of these assays leverage the interaction between RBD and angiotensin-converting enzyme 2 in their lateral flow development. The COVID-19 Nab-test was studied in a cohort of 79 individuals infected with SARS-CoV-2 and was found to strongly correlate with a microneutralization assay (NT50 ≥1:40), *R*^2^ = 0.8 (*P* < 0.0001), and a reported sensitivity of 79.1% to 99.8% [33]. The quantitative LFA described by Lake et al was compared to neutralization assays from individuals infected and vaccinated and found to have an ROC AUC of 98% at NT50 ≥1:320, with a sensitivity of 90% at an assay cutoff of density units < 263,000 (n = 38 samples) [34]. These assays, like our platform, are based on RBD antigens, show good correlation with NAb titers, and are a promising start in the development of a rapid, quantitative assay surrogate for NAb levels and, therefore, humoral protection against COVID-19 disease. Compared with prior studies, the sqLFA assay reported here used a substantially larger set of samples derived from natural human infection and performed a more granular analysis, reporting sensitivity and specificity across a range of potential NT50 targets.

Since optimal NT50 protective NAb titers as a CoP are still being determined, the present study tested a variety of NT50 cutoffs as a less biased analysis of assay performance.

The main limitation of this type of study is the lack of guidance from the scientific community on what is a good target sensitivity and specificity for a rapid point-of-care test to detect CoP. There has yet to be a longitudinal study evaluating SARS-CoV-2 correlates of protection in a natural infection cohort. In this regard, the lack of widely available functional neutralizing antibody assays may hinder progress in precise immune correlates research and downstream clinical implementation. One could envision a point-of-care assay such as this to enable larger-scale immune protection studies, using a complex assay such as NT50 as a gold standard and establishing targets of protection that can be correlated with the more deployable sqLFA. Although resource limitations prevented inclusion of more recent SARS-CoV-2 variants in this study, recent publications on contemporary strains reveal ongoing strong correlations between RBD-binding antibodies and NAbs [35, 36]. In the event of variant emergence, the sqLFA can be rapidly updated with new variant-specific antigens. Additionally, the sqLFA is expandable to allow inclusion of RBD-targeting IgA and IgM on the LFA strip. Including these antibody isotypes is important as they may contribute to viral neutralization, especially in the first few months post infection, and may further improve sqLFA sensitivity and specificity.

Another limitation is that, to our knowledge, ours is the largest sample size used to study correlations between point-of-care sqLFA results and NT50 titers. However, the cohort may not represent the full range of patient demographics that may benefit from clinical applications of this assay, such as immunocompromised patients who are generally underrepresented (see our prior publication for demographic information on this cohort) [19]. Nonetheless, the relatively low material cost and time investment required to perform the sqLFA point-of-care test make it an attractive adjuvant assay in future larger vaccine efficacy studies that may include NAb measurements to identify a NAb NT50 correlate titer and could simultaneously test the performance of a semi-quantitative point-of-care test such as the BioMedomics sqLFA.

Given ongoing transmission of SARS-CoV-2 variants, as well as known waning antibody levels to both natural infection and vaccination [37], the development of rapid quantitative assays to identify individuals at risk for re-infection is critical. In the SARS-CoV-2 mRNA-vaccinated healthcare worker study by Bergwerk et al, individuals in the cohort with breakthrough infections had a 6- to 7-fold lower mean NAb titer than those who had not experienced a vaccine breakthrough infection [13]. Furthermore, it has been shown that standard 2-dose [38] or even 3-dose [39] mRNA vaccines in solid-organ transplant recipients may not produce an adequate immune response. Current vaccine guidelines recommend annual COVID-19 vaccine boosters for the elderly and those with specific medical conditions or who are immunocompromised [40]. As the landscape of cost-benefit analyses of repeated vaccination for individuals without these major risk factors evolves, having a reliable, easy-to-use, rapid clinical assay that detects protective levels of NAbs and identifies individuals who may need additional SARS-CoV-2 vaccination is a missing yet critical component of current management of SARS-CoV-2 transmission and disease. Readily adaptable antigen-based platforms should be clinically available to be able to evaluate an individual’s degree of vulnerability to disease.

## SUPPLEMENTARY DATA

Supplementary materials are available at the *Pathogens and Immunity* website. Supplementary data may be provided by the authors to benefit the reader. Supplementary data are not copyedited and are the sole responsibility of the authors. Questions or comments related to supplementary materials should be addressed to the corresponding author.
