# Supplementary material for: A Novel Adaptive Platform for Rapid, Simple Flow-Based Antibody Detection Devices Predicts NAb Levels to SARS-CoV-2
Source: Pathog Immun. 2026 Feb 16;11(1):96–110. doi: 10.20411/pai.v11i1.910 (PMC12928283; doi:10.20411/pai.v11i1.910)
Supplement: Supplementary Table and Figure [file pai-11-096-s01.pdf]

PATHOGENS AND IMMUNITY
IN ASSOCIATION WITH CASE WESTERN RESERVE UNIVERSITY AND THE RICHARD J. FASENMYER FOUNDATION

Supplementary Table 1. Heat Index Correlation Figure (Figure 2C) Sample Sizes

	sqLFA	WA-1 NT50	RBD IgG	DiaSorin S IgG	Abbott N IgG	PSV NT50
**sqLFA**	268	268	268	94	158	262
**NT50**	268	268	268	94	158	262
**RBD IgG**	268	268	268	94	158	262
**DiaSorin S IgG**	94	94	94	94	57	92
**Abbott N IgG**	158	158	158	57	158	157
**PSV NT50**	262	262	262	92	157	262

Number of COVID-19 recovered participant samples included in each analysis.

Supplementary Table 2. Sensitivity and Specificity of qLFA for Detecting Neutralizing Antibodies at Additional NT50 Cutoffs of Interest

qLFA cutoff	AUC	Sensitivity (%)	95% CI	Specificity (%)	95% CI
**NT50 ≥ 1:40**	0.93, p<0.0001				
>80 (manufacturer's cutoff)		97	93.2-98.3	46	34.0-58.3
>457		80	74.2-85.0	90	80.2-95.4
**NT50 ≥ 1:54** >457	0.89, p <0.0001	82	75.5-86.4	82	72.0-89.3
**NT50 ≥ 1:160**	0.85, p <0.0001				
>563		85	78.2-90.2	69	60.7-76.1
**NT50 ≥ 1:640**	0.82, p<0.0001				
>872		90	76.4-96.0	67	60.5-72.6
**NT50 ≥ 1:1024**	0.71, p=0.0016				
>872		80	58.4-91.9	62	55.5-67.5

ROC analysis results for each NT50 cutoff, showing the sqLFA cutoff with the best Youden's J statistic to maximize both Sensitivity and Specificity. 95% CI = 95% confidence intervals.

Markmann AJ, Bhowmik DR, Jiang B, Vanna UP, Van Hoy M, Wang F, Hou YJ, Margolis DM, Baric RS, de Silva AM, Bartelt LA. A Novel Adaptive Platform for Rapid, Simple Flow-Based Antibody Detection Devices Predicts NAb Levels to SARS-CoV-2. *Pathogens and Immunity*. 2025;11(1):96-110. doi: 10.20411/pai.v11i1.910

PATHOGENS AND IMMUNITY
IN ASSOCIATION WITH CASE WESTERN RESERVE UNIVERSITY AND THE RICHARD J. FASENMYER FOUNDATION

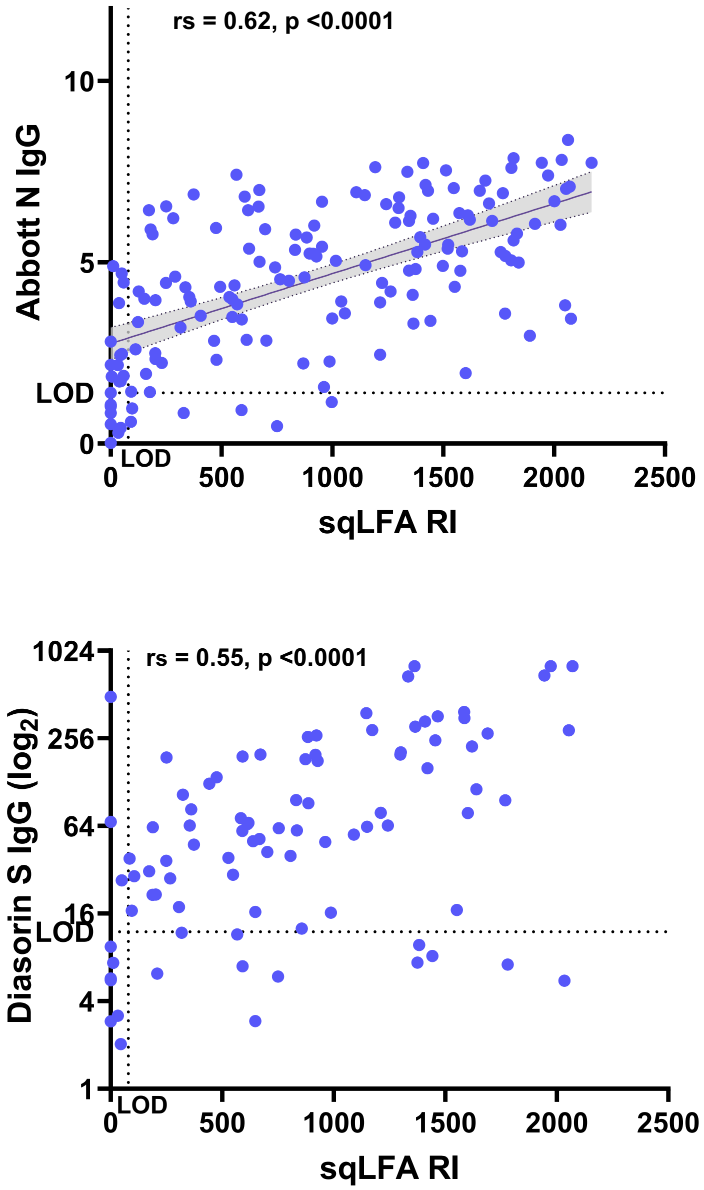

Supplementary Figure 1. **Correlation plots of sqLFA with other diagnostic and functional assays.** A) Abbott Nucleocapsid antibody assay correlation plot with sqLFA, grey shaded area is 95% Confidence Intervals for simple linear regression analysis. B) Diasorin Spike antibody assay correlation plot with sqLFA.

Markmann AJ, Bhowmik DR, Jiang B, Vanna UP, Van Hoy M, Wang F, Hou YJ, Margolis DM, Baric RS, de Silva AM, Bartelt LA. A Novel Adaptive Platform for Rapid, Simple Flow-Based Antibody Detection Devices Predicts NAb Levels to SARS-CoV-2. *Pathogens and Immunity*. 2025;11(1):96-110. doi: 10.20411/pai.v11i1.910
